# Factors associated with postpartum depression among Myanmar women in Yangon: A cross-sectional study

**DOI:** 10.1016/j.heliyon.2024.e33425

**Published:** 2024-06-25

**Authors:** Kaung Myat Wyunn, Zin Zin Than Wai, Khin Ei Ei Swe, Souphalak Inthaphatha, Kimihiro Nishino, Eiko Yamamoto

**Affiliations:** a Department of Healthcare Administration, Nagoya University Graduate School of Medicine, Nagoya, Japan; b Department of Medical Services, Ministry of Health, Nay Pyi Taw, Myanmar; c Yangon General Hospital, Yangon, Myanmar; d North Okkalapa General and Teaching Hospital, Yangon, Myanmar; e Asian Satellite Campuses Institute, Nagoya University, Nagoya, Japan

**Keywords:** Edinburgh postnatal depression scale, Myanmar, Postpartum depression

## Abstract

**Background:**

Postpartum depression (PPD) is non-psychotic depressive illness after childbirth, and PPD affects the health and social status of mothers and their children. This study aims to identify the prevalence of suspected PPD and associated factors among women in Yangon, Myanmar.

**Methods:**

This is a cross-sectional study of 552 women at 6–8 weeks postpartum who visited at North Okkalapa General and Teaching Hospital for postnatal care from September to October 2022. Socio-demographic factors, obstetric and infant factors, family support, and medical history were collected using a structured questionnaire. Myanmar version of the Edinburgh Postnatal Depression Scale (EPDS) was used for screening PPD, and all women were divided into suspected PPD (EPDS ≥10) and non-suspected PPD (EPDS <10). Chi-square and Fisher's exact tests were used to compare the characteristics of women between suspected PPD and non-suspected PPD. Logistic regression analysis was preformed to identify the factors associated with suspected PPD.

**Results:**

The mean age of the 552 women was 27.9 years and 176 women (31.9 %) were categorized into suspected PPD. Factors associated with suspected PPD were living in a nuclear family (adjusted odds ratio (AOR) = 2.04, 95 % confidence interval (CI) 1.10–3.77), feeling insufficient income (AOR = 2.15, 95 % CI 1.09–4.25), unplanned pregnancy (AOR = 2.76, 95 % CI 1.47–5.16), less than eight ANC visits (AOR = 3.29, 95 % CI 1.74–6.23), low birth weight of the last baby (AOR = 5.69, 95 % CI 1.17–27.71), insufficient support from partners (AOR = 11.28, 95 % CI 5.62–22.63), parents (AOR = 3.83, 95 % CI 2.08–7.04), and parents-in-law (AOR = 2.00, 95 % CI 1.06–3.76), and depressive symptoms during the last pregnancy (AOR = 3.08, 95 % CI 1.52–6.26).

**Conclusion:**

The prevalence of suspected PPD was 31.9 % among 6-8-week postpartum women in Yangon. Strengthening maternal health programs and providing education about postpartum psychiatric problems is essential to improve maternal psychological well-being.

## Highlights

• The prevalence of suspected postpartum depression (PPD) was 31.9 % in Yangon.
• Insufficient income and family support were associated with suspected PPD.
• Depression during pregnancy was associated with suspected PPD.
• Unplanned pregnancy, less antennal care, and low birth weight were risk factors.

## 1 Introduction

Postpartum depression (PPD) is a non-psychotic depressive illness after childbirth [1]. Symptoms of PPD gradually appear around six weeks after delivery and can last for a year [2]. PPD should be detected in the early stage because it negatively affects the health and social conditions of mothers as well as their children [2,3]. Compared to mothers without PPD, mothers with PPD are more likely to have children with psychiatric and developmental disorders and infectious diseases [3,4]. According to a systematic review including 565 studies in 80 different countries and areas, the prevalence of PPD was estimated to be 17.2 % worldwide in 2021 [5]. Previous studies have suggested that the prevalence of PPD may be higher in developing countries than in developed countries [2,4]. PPD is a treatable disorder; however, in most developing countries, women with PPD do not receive appropriate care or treatment since mental health care services are not included in maternal and child health programs [6].

Factors associated with PPD are reported to be socio-demographic factors (low education, being a single mother, having financial problems), maternal and child factors (unplanned pregnancy, preterm delivery, no or fewer visits for antenatal care (ANC) and postnatal care (PNC), living far from health facilities, baby's complication, and unwanted sex of a baby), interpersonal relationships and poor support from partners, parents, parents-in-law, and friends, and a history of depression [3,[7], [8], [9], [10], [11], [12], [13]]. In addition, the COVID-19 pandemic negatively influenced the mental status of postpartum women because the prevalence of PPD was higher during the pandemic compared to that in 2019 or before [[14], [15], [16]].

Myanmar is a lower middle-income country in Southeast Asia with a population of over 51 million [17]. Over the decades, the health service system has been developed to meet the needs of the people [18]. However, the mental health service is insufficient. There are only two psychiatric hospitals in the country, and only public hospitals with 200 beds or more have mental health departments [19]. There have been only a few studies on PPD among Myanmar women and most studies included Myanmar women who lived in Thailand as refugees or migrants [8,11,20]. Only one study was conducted in a rural area of Myanmar and the prevalence of PPD was 31.8 % among 220 postpartum women within six months [11]. Unplanned pregnancy, preterm delivery, less than four ANC visits, and residence far from health facilities were associated with PPD [11]. However, to our knowledge, no paper has been published on PPD in urban areas of Myanmar. Many policies and strategies have been established to improve maternal and child health, but there has been no policy on PPD due to a lack of information on maternal mental health in Myanmar [21]. Therefore, it is necessary to recognize the current mental disorders among postpartum women, especially in urban areas of Myanmar. This study aimed to identify the prevalence and factors associated with PPD among women who visited a tertiary hospital in Yangon.

## 2 Methods

### 2.1 Study design and participants

This cross-sectional study was conducted at North Okkalapa General and Teaching Hospital in Yangon, Myanmar, a tertiary hospital with 800 sanctioned beds and affiliated with the University of Medicine 2, Yangon. It has 35 departments, including obstetrics, gynecology, pediatrics, and mental health. According to the statistical data at the hospital, approximately 100 women visited the PNC and expanded immunization program (EPI) clinics every week in July 2022. All women who had childbirth 6–8 weeks previously and visited the hospital to attend the PNC and EPI clinics from September to October 2022 were included in this study. Women younger than 18 years old, who had multiple births, who did not answer all questions on the survey form, who could not understand the Myanmar language, or whose last child died were excluded. The sample size was calculated based on the predicted prevalence of PPD (31.8 %) in Myanmar with a reliability coefficient of 1.96 at the 95 % confidence interval (CI) and a margin of error of 0.04 [11]. The sample size was 573 after adding the non-response rate (10 %) to the minimum required sample size. In this study, a total of 580 women participated, but 28 were excluded due to incomplete survey forms (n = 6), twin deliveries (n = 2), and death of the last child (n = 20). Finally, this study comprises the data of 552 women.

### 2.2 Data collection

A structured questionnaire was developed to collect the socio-demographic characteristics of postpartum women and their partners, obstetric and infant factors, support from family and friends, and the medical history of postpartum women (Supplementary Table 1). The questionnaire was revised after a pretest was conducted with 20 postpartum women at the hospital on September 1, 2022. Seven healthcare workers of the hospital including a deputy director, a junior obstetrician, two head nurses, and three senior nurses, interviewed 580 postpartum women using the questionnaire, while the women completed the Edinburgh Postnatal Depression Scale (EPDS) by themselves unless they had a problem with reading. The seven healthcare workers were trained by the principal investigator (Dr. Kaung Myat Wyunn) about data collection using the questionnaire and not forcing participants to respond. Private rooms were used for the interview to protect the participants’ privacy. Written informed consent was obtained from each participant before the interview. In addition to the interviews, ANC logbooks, and child weight monitoring records were used to collect information about the last pregnancy of postpartum women and their last babies.

### 2.3 Socio-demographic factors

Socio-demographic characteristics of postpartum women included age, residence, ethnicity, religion, marital status, occupation, education, partner's occupation and education, type of family, monthly household income, and feeling about household income. According to the mean age of the participants (27.9 years), age was divided into two categories (≤28 years and >28 years). Their residences were divided into Yangon Region and others (states and regions other than Yangon Region). The ethnic groups were categorized according to the ethnicity classification of the Myanmar government and divided into Myanmar and others (Kachin, Kayah, Karen, Chin, Mon, Rakhine, and Shan) [22]. The participants' religions were divided into two groups, namely Buddhism and others (Islam, Christianity, Hinduism, and atheism). The marital status was divided into married and others (single, living together but not married, divorced, and widowed). The educational status was determined by the highest grade they received and was divided into two categories (high school or less and diploma/bachelor degree or more). The women chose either living independently in a nuclear family or living with their parents or parents-in-law in an extended family. Monthly household income was the total monthly income of all members of the women's household, and divided into two categories (≤700,000 Myanmar Kyat (MMK) and >700,000 MMK) based on the mean (695,000 MMK). The women were also asked if they felt that the household income was enough to cover the family's living expenses, and responses were “yes” or “no”.

### 2.4 Obstetrics and infant factors

Obstetrics and infant factors included parity, miscarriage history, and factors related to the last pregnancy and childbirth of the women, such as planned pregnancy, ANC visits, complications during the pregnancy, mode and place of delivery, birth assistant, gestational age, birth weight, sex, complications of the last baby, and the breastfeeding status. Parity was divided into one and two or more. The women were asked whether their last pregnancy was planned or not. ANC visits during the last pregnancy were categorized into two groups (<8 visits and ≥8 visits) according to World Health Organization's recommendation [23]. The gestational age of the last childbirth was divided into two categories (≤36 weeks and ≥37 weeks) based on the definitions of preterm and term delivery. Based on the definition of low birth weight (LBW), the birth weight of the last baby was categorized into <2,500g (LBW) and ≥2,500g [24]. Interviews, ANC logbooks, and child weight monitoring records were used to collect information on complications during the last pregnancy, mode, and place of delivery, the birth assistant, birth weight, and complications of the last baby. The participants were asked about the sex of their last baby and if the sex was the same as they desired before the delivery.

### 2.5 Support from family and friends

The women were asked if they had support from their partners, parents, parents-in-law, and friends during the last pregnancy and after the last childbirth. The support was assessed using a five-point Likert scale; very good, good, neutral, bad, and very bad. The responses were then categorized into yes (very good and good) and no (neutral, bad, and very bad). Women who were single, widowed, or divorced and whose parents or parents-in-law had died were categorized into “no”.

### 2.6 Medical history

In terms of medical history, the women were asked if they had ever had depressive symptoms that lasted more than two weeks before and during the last pregnancy. Depressive symptoms included feeling sadness, hopelessness, crying, noticeable loss of interest, having a trouble with eating or sleeping, feeling tired, feeling worthless almost all the time, and having frequent thoughts of death.

### 2.7 EPDS

Cox et al. developed the EPDS as a tool to screen PPD, and it has been used in community-based and hospital-based studies in many countries in the world [[25], [26], [27]]. EPDS includes 10 questions that ask women to rate how they have felt in the past seven days, and each item has four points on the Likert scale (0–3 points), resulting in a total scale of 0–30. Myanmar version of EPDS, which was validated and utilized in previous studies, was used in this study after confirming its comprehensibility for Myanmar women in the pretest [11,20,27]. In accordance with a previous study on validation of the Myanmar version of EPDS, a cut-off score of 10 or higher was used to define women with suspected PPD in this study [20]. All women were divided into suspected PPD (EPDS ≥10) and non-suspected PPD (EPDS <10). Women were encouraged to answer all questions completely and requested not to share their answers with others [25].

### 2.8 Statistical analysis

Statistical analyzes were performed using SPSS, version 25 (IBM SPSS Inc, New York, USA). Chi-square test and Fisher's exact test were performed to compare the characteristics of women between suspected PPD and non-suspected PPD. Multiple logistic regression analysis was performed to identify factors associated with suspected PPD using variables that had statistically significant differences by the chi-square test or Fisher's exact test. The CI was set at 95 %, and a P-value of less than 0.05 was considered statistically significant.

### 2.9 Ethical issues

This study was approved by the Ethics Committee of the Myanmar Ministry of Health (approval number: NOGTH/2/2022). Written informed consent was obtained from each participant. Participants were not requested to answer their names in this study, and all information was kept confidential.

## 3 Results

### 3.1 Socio-demographic factors of the participants

A total of 552 women who visited PNC and EPI clinics of the North Okkalapa General and Teaching Hospital in Yangon were included in the study. The mean age of the 552 women was 27.9 years (standard deviation (SD), 4.8) and the age ranged from 18 to 43 years. Most women (n = 534, 96.7 %) were from the Yangon region, belonged to Myanmar ethnic group (n = 405, 73.4 %), and believed in Buddhism (n = 418, 75.7 %) (Table 1). Most women were married (n = 525, 95.1 %), housewives (n = 229, 41.5 %), and had education at high school level or lower (n = 327, 59.2 %). Concerning their partners’ characteristics, most partners were company and government employees (n = 277, 50.2 %) and had education at high school level or lower (n = 307, 56.5 %). The proportion of the women who lived in nuclear families was 44.2 % (n = 244). Regarding monthly household income, 72.6 % of the women (n = 401) had 700,000 MMK (approximately 332 USD) or less, and 29.3 % (n = 162) reported that household income was not enough to cover their living costs.

**Table 1:** Socio-demographic characteristics of 552 postpartum women.

Variables	Total (N = 552)	EPDS <10 (N = 376)	EPDS ≥10 (N = 176)	P-value^g^
	N (%)	n (%)	n (%)	
**Age group (years)**				0.009
≤28	314 (56.9)	228 (60.6)	86 (48.9)	
>28	238 (43.1)	148 (39.4)	90 (51.1)	
**Residence**				0.704
Yangon region	534 (96.7)	363 (96.5)	171 (97.2)	
Others	18 (3.3)	13 (3.5)	5 (2.8)	
**Ethnic group**				0.314
Myanmar	405 (73.4)	271 (72.1)	134 (76.1)	
Others^a^	147 (26.6)	105 (27.9)	42 (23.9)	
**Religion**				0.877
Buddhism	418 (75.7)	284 (75.5)	134 (76.1)	
Others^b^	134 (24.3)	92 (24.5)	42 (23.9)	
**Marital status**				<0.001
Married	525 (95.1)	368 (97.9)	157 (89.2)	
Others^c^	27 (4.9)	8 (2.1)	19 (10.8)	
**Occupation**				0.285
Housewife	229 (41.5)	157 (41.8)	72 (40.9)	
Manual labor	126 (22.8)	79 (21.0)	47 (26.7)	
Others^d^	197 (35.7)	140 (37.2)	57 (32.4)	
**Education**				0.046
High school or lower	327 (59.2)	212 (56.4)	115 (65.3)	
Diploma/bachelor or higher	225 (40.8)	164 (43.6)	61 (34.7)	
**Partner's occupation**				0.680
Company/government employees	277 (50.2)	193 (51.3)	84 (47.7)	
Manual labor	139 (25.2)	91 (24.2)	48 (27.3)	
Others^e^	136 (24.6)	92 (24.5)	44 (25.0)	
**Partner's education**^f^				0.998
High school or lower	307 (56.5)	212 (56.5)	95 (56.5)	
Diploma/bachelor or higher	236 (43.5)	163 (43.5)	73 (43.5)	
**Type of family**				0.047
Nuclear family	244 (44.2)	177 (47.1)	67 (38.1)	
Extended family	308 (55.8)	199 (52.9)	109 (61.9)	
**Monthly household income (MMK)**				0.004
≤700,000	401 (72.6)	259 (68.9)	142 (80.7)	
>700,000	151 (27.4)	117 (31.1)	34 (19.3)	
**Income for household living**				<0.001
Enough	390 (70.7)	298 (79.3)	92 (52.3)	
Not enough	162 (29.3)	78 (20.7)	84 (47.7)	

EPDS, Edinburgh Postnatal Depression Scale; MMK, Myanmar Kyats (1 USD = 2100.08 MMK, as of February 20, 2022).

a Others are Kachin, Kayah, Karen, Chin, Mon, Rakhine, and Shan.

b Others include Islam, Christianity, Hinduism, and atheism.

c Others include single, living together but not married, divorced, and widowed.

d Others include student, company/government employees, own business, and job outside Myanmar.

e Others include student, no job, unknown, and no partner.

f 543 women were included because nine women did not answer the question.

g Chi-square test was used.

### 3.2 Obstetrics and infant factors of the participants

The proportion of women who had only one child was 31.5 % (n = 174), and a history of miscarriage was found in 21.2 % of the women (n = 117) (Table 2). Most women planned their last pregnancy (n = 332, 60.1 %), and had fewer than eight ANC visits (n = 286, 51.8 %). Of all the women, 30.1 % (n = 166) experienced complications during the last pregnancy. Most women had vaginal deliveries, but 23.7 % of women (n = 131) underwent a cesarean section. Of all the 552 women, 535 women (96.9 %) had the last childbirth at hospitals or health centers, and 538 women (97.5 %) received assistance from doctors, nurses, and midwives for the last childbirth. Most women gave a full-term birth, but 78 women (14.1 %) and 69 women (12.5 %) had pre-term deliveries and LBW babies (<2,500g), respectively. Regarding the last baby's sex, 279 women (50.5 %) responded that it was the same as they desired. The percentage of women whose babies were hospitalized after birth was 32.4 % (n = 179). Regarding the feeding method, 471 women (87.9 %) gave only breast milk, 21 women (3.9 %) gave only formula, and 44 women (8.2 %) used both breast milk and formula.

**Table 2:** Obstetrics and infant factors of 552 postpartum women.

Variables	Total (N = 552)	EPDS <10 (n = 376)	EPDS ≥10 (n = 176)	P-value^a^
	N (%)	n (%)	n (%)	
**Parity**				0.282
1	174 (31.5)	124 (33.0)	50 (28.4)	
≥2	378 (68.5)	252 (67.0)	126 (71.6)	
**History of miscarriage**				0.336
No	435 (78.8)	292 (77.7)	143 (81.2)	
Yes	117 (21.2)	84 (22.3)	33 (18.8)	
**Planned pregnancy**				<0.001
Yes	332 (60.1)	274 (72.9)	58 (33.0)	
No	220 (39.9)	102 (27.1)	118 (67.0)	
**ANC visits during the last pregnancy**				<0.001
<8 visits	286 (51.8)	152 (40.4)	134 (23.9)	
≥8 visits	266 (48.2)	224 (59.6)	42 (76.1)	
**Complications during the last pregnancy**				<0.001
No	386 (69.9)	286 (76.1)	100 (56.8)	
Yes	166 (30.1)	90 (23.9)	76 (43.2)	
**Mode of delivery**				0.047
Vaginal delivery	421 (76.3)	296 (78.7)	125 (71.0)	
Caesarean section	131 (23.7)	80 (21.3)	51 (29.0)	
**Place of delivery**				0.173
Hospital/health center	535 (96.9)	367 (97.6)	168 (95.5)	
Home	17 (3.1)	9 (2.4)	8 (4.5)	
**Birth assistant**				0.001^b^
Doctor/nurse/midwife	538 (97.5)	372 (98.9)	166 (94.3)	
Traditional birth attendant	14 (2.5)	4 (1.1)	10 (5.7)	
**Gestational age**				<0.001
≤36 weeks	78 (14.1)	32 (8.5)	46 (26.1)	
≥37 weeks	474 (85.9)	344 (91.5)	130 (73.9)	
**Birth weight of the last baby**				<0.001
<2,500g	69 (12.5)	22 (5.9)	47 (26.7)	
≥2,500g	483 (87.5)	354 (94.1)	129 (73.3)	
**Sex of the last baby**				<0.001
Same as desire	279 (50.5)	218 (58.0)	61 (34.7)	
Different from desired	273 (49.5)	158 (42.0)	115 (65.3)	
**Complications of the last baby**				<0.001
No	373 (67.9)	289 (76.9)	84 (47.7)	
Yes	179 (32.4)	87 (23.1)	92 (52.3)	
**Breastfeeding status**				0.055
Breast milk only	471 (87.9)	335 (90.1)	136 (82.9)	
Baby formula only	21 (3.9)	13 (3.5)	8 (4.9)	
Breast milk and baby formula	44 (8.2)	24 (6.5)	20 (12.2)	

EPDS, Edinburgh Postnatal Depression Scale; ANC, antenatal care.

a Chi-square test was used except in the variable where Fisher's exact test was applied.

b Fisher's exact test was used.

### 3.3 Support from family and friends and medical history of the participants

During the last pregnancy and delivery, 72.6 % of the women (n = 401) received support from their partners, 60.7 % (n = 335) from their parents, 47.5 % (n = 262) from their parents-in-law, and 53.8 % (n = 297) from their friends (Table 3). Of the 552 women, 77 women (13.9 %) had depressive symptoms for more than two weeks before the last pregnancy, while 109 women (19.7 %) had the symptoms during the last pregnancy (Table 3).

**Table 3:** Support from family and friends and medical history of 552 postpartum women.

Variables	Total (N = 552)	EPDS <10 (n = 376)	EPDS ≥10 (n = 176)	P-value^a^
	N (%)	n (%)	n (%)	
**Support from partner**				<0.001
Yes	401 (72.6)	343 (91.2)	58 (33.0)	
No	151 (27.4)	33 (8.8)	118 (67.0)	
**Support from parents**				<0.001
Yes	335 (60.7)	279 (74.2)	56 (31.8)	
No	217 (39.3)	97 (25.8)	120 (68.2)	
**Support from parents-in-law**				<0.001
Yes	262 (47.5)	229 (60.9)	33 (18.7)	
No	290 (52.5)	147 (39.1)	143 (81.3)	
**Support from friends**				<0.001
Yes	297 (53.8)	235 (62.5)	62 (35.2)	
No	255 (46.2)	141 (37.5)	114 (64.8)	
**Depressive symptoms before the last pregnancy**				<0.001
No	475 (86.1)	348 (92.6)	127 (72.2)	
Yes	77 (13.9)	28 (7.4)	49 (27.8)	
**Depressive symptoms during the last pregnancy**				<0.001
No	443 (80.3)	337 (89.6)	106 (60.2)	
Yes	109 (19.7)	39 (10.4)	70 (39.8)	

EPDS, Edinburgh Postnatal Depression Scale.

a Chi-square test was used.

### 3.4 Factors associated with suspected PPD

**Fig. 1:**
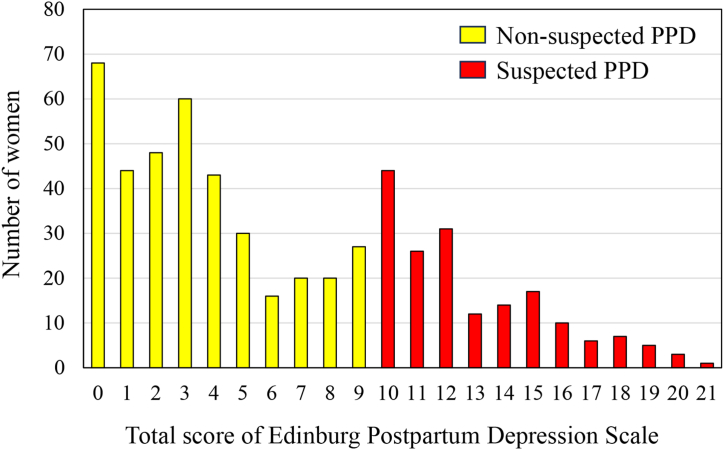
Distribution of total score of Edinburgh Postnatal Depression Scale among 552 women at 6–8 weeks postpartum in Myanmar. PPD, postpartum depression.

The total score of EPDS of the 552 women ranged from 0 to 21, with a mean of 6.4 (SD, 5.2). There were 176 women (31.9 %) whose EPDS was 10 or higher, and they were defined as suspected PPD in this study (Fig. 1). The results of chi-square test and Fisher's exact test showed that the distribution of age, marital status, educational level, type of family, monthly household income, feeling about household income, unplanned pregnancy, ANC visits, complications during the last pregnancy, mode of delivery, birth assistants, gestational age, birth weight of the last baby, sex of the last baby, complication of the last baby, support from the partners, parents, parents-in-law, and friends during the antenatal and postnatal periods, history of depressive symptoms for more than two weeks before and during the last pregnancy were significantly different between women with suspected PPD and those with non-suspected PPD (Table 1, Table 2, Table 3).

Multivariate logistic regression analysis on suspected PPD was performed, including 21 variables that showed significant differences in the prevalence of suspected PPD by chi-square or Fisher's exact tests (Table 4). Of the socio-demographic factors, living in a nuclear family (adjusted odds ratio (AOR) = 2.04, 95 % CI 1.10–3.77, P = 0.023) compared to living in an extended family and feeling not enough income for household living (AOR = 2.15, 95 % CI 1.09–4.25, P = 0.028) were associated with suspected PPD. Of the obstetrics and infant factors, having an unplanned pregnancy (AOR = 2.76, 95 % CI 1.47–5.16, P = 0.002), having less than eight ANC visits during the last pregnancy (AOR = 3.29, 95 % CI 1.74–6.23, P < 0.001), and having a baby with LBW (AOR = 5.69, 95 % CI 1.17–27.71, P = 0.031) were associated with suspected PPD. Women who had no support from their partners (AOR = 11.28, 95 % CI 5.62–22.63, P < 0.001), their parents (AOR = 3.83, 95 % CI 2.08–7.04, P < 0.001), and their parents-in-law (AOR = 2.00, 95 % CI 1.06–3.76, P = 0.032), and women who had depressive symptoms for more than two weeks during the last pregnancy (AOR = 3.08, 95 % CI 1.52–6.26, P = 0.002) were also associated with suspected PPD.

**Table 4:** Binary and multivariate logistic regressions on suspected postpartum depression.

Variables	Unadjusted		Adjusted^a^	
	OR (95%CI)	P-value	AOR (95%CI)	P-value
**Age group (years)**				
≤28	Reference		Reference	
>28	1.61 (1.12–2.31)	0.009	1.03 (0.56–1.90)	0.938
**Marital status**				
Married	Reference		Reference	
Others^b^	5.58 (2.39–12.98)	<0.001	1.34 (0.37–4.87)	0.660
**Education**				
High school or lower	1.46 (1.01–2.11)	0.046	1.48 (0.77–2.81)	0.239
Diploma/bachelor or higher	Reference		Reference	
**Type of family**				
Nuclear family	1.45 (1.00–2.09)	0.048	2.04 (1.10–3.77)	0.023
Extended family	Reference		Reference	
**Monthly household income (MMK)**				
≤700,000	1.89 (1.22–2.91)	0.004	1.33 (0.61–2.87)	0.471
>700,000	Reference		Reference	
**Income for household living**				
Enough	Reference		Reference	
Not enough	3.49 (2.37–5.17)	<0.001	2.15 (1.09–4.25)	0.028
**Planned pregnancy**				
Yes	Reference		Reference	
No	5.47 (3.71–8.06)	<0.001	2.76 (1.47–5.16)	0.002
**ANC visits during the last pregnancy**				
<8 visits	4.70 (3.14–7.04)	<0.001	3.29 (1.74–6.23)	<0.001
≥8 visits	Reference		Reference	
**Complications during the last pregnancy**				
No	Reference		Reference	
Yes	2.42 (1.65–3.54)	<0.001	1.42 (0.73–2.76)	0.300
**Mode of delivery**				
Vaginal delivery	Reference		Reference	
Caesarean section	1.51 (1.00–2.27)	0.048	1.39 (0.68–2.83)	0.367
**Birth assistant**				
Doctor/nurse/midwife	Reference		Reference	
Traditional birth attendant	5.60 (1.73–18.12)	0.004	2.50 (0.20–31.27)	0.478
**Gestational age**				
≤36 weeks	3.80 (2.32–6.24)	<0.001	2.47 (0.54–11.29)	0.242
≥37 weeks	Reference		Reference	
**Birth weight of the last baby**				
<2,500g	5.86 (3.40–10.11)	<0.001	5.69 (1.17–27.71)	0.031
≥2,500g	Reference		Reference	
**Sex of the last baby**				
Same as desire	Reference		Reference	
Different from desired	2.60 (1.79–3.77)	<0.001	1.64 (0.90–2.99)	0.110
**Complications of the last baby**				
No	Reference		Reference	
Yes	3.64 (2.49–5.32)	<0.001	2.12 (1.09–4.14)	0.635
**Support from partner**				
Yes	Reference		Reference	
No	9.90 (5.45–17.97)	<0.001	11.28 (5.62–22.63)	<0.001
**Support from parents**				
Yes	Reference		Reference	
No	6.16 (4.16–9.13)	<0.001	3.83 (2.08–7.04)	<0.001
**Support from parents-in-law**				
Yes	Reference		Reference	
No	6.75 (4.39–10.29)	<0.001	2.00 (1.06–3.76)	0.032
**Support from friends**				
Yes	Reference		Reference	
No	3.07 (2.11–4.45)	<0.001	1.74 (0.95–3.18)	0.073
**Depressive symptoms before the last pregnancy**				
No	Reference		Reference	
Yes	4.80 (2.89–7.96)	<0.001	1.53 (0.64–3.65)	0.339
**Depressive symptoms during the last pregnancy**				
No	Reference		Reference	
Yes	5.71 (3.65–8.94)	<0.001	3.08 (1.52–6.26)	0.002

OR, odds ratio; AOR, adjusted odds ratio; CI, confidence interval; ANC, antenatal care.

a Adjusted for all variables listed in the table.

b Others include single, living together but not married, divorced, and widowed.

## 4 Discussion

In this study, the prevalence of suspected PPD was 31.9 % among 6–8 weeks postpartum women in Yangon. A previous study conducted in rural areas of Yangon in 2020 reported that the prevalence of PPD was 31.8 %, but the cut-off score of EPDS for PPD was 13 or above [11]. When the same cut-off score (≥13) is used, the prevalence of suspected PPD is 13.6 % in the present study. These results suggest that the prevalence in the previous study might be higher than the current study, and it may be because it was conducted during the first wave of the COVID-19 pandemic in Myanmar. The prevalence of suspected PPD during this study was the same as that in the neighboring countries, such as Lao PDR (31.8 % using EPDS ≥10) and Bangladesh (34.0 % using EPDS ≥10) [9,28]. The prevalence was still higher than that of other Asian countries: 7.8 % in Sri Lanka's provinces using EPDS ≥10, 17.4 % in a hospital-based study in Thailand using EPDS ≥11, and 11.4 % at maternity hospitals in Shanghai, China using EPDS ≥10 [[29], [30], [31]]. It has been said that programs for maternal and child health in Myanmar must increase services to support early diagnosis and treatment of postnatal mental illnesses.

In this study, women who felt their family income was insufficient, had unplanned pregnancies, and had LBW babies were associated with suspected PPD. These results were consistent with the results of many previous studies and suggest that pregnant and postpartum women may be at risk of developing PPD due to negative feelings and high emotional stress [9,11,[32], [33], [34], [35]]. Healthcare providers should be aware of psychological discomfort experienced by mothers and refer them to psychological counseling to prevent PPD [33,35]. Family planning can prevent unintended pregnancies; women and their partners should make a precise family plan, along with a financial plan, before getting pregnant [36]. In Myanmar, public health facilities provide free family planning services [37]; however, the strategy needs to be enhanced to reduce unplanned pregnancies.

This study revealed that suspected PPD was associated with women who had less than eight ANC visits during the last pregnancy, which was similar to the results of previous studies in Myanmar and other countries [11,38]. It may be because pregnant women who did not have at least eight ANC visits may not have received adequate prenatal counseling on pregnancy-related problems [23]. When pregnant women visit ANC frequently, they have continuous counseling and support from healthcare providers, which can reduce antenatal anxiety [39]. Therefore, it is important for ANC providers to understand PPD in order to provide education about PPD and identify women who need psychosocial support.

In this study, women who lived in a nuclear family were more likely to have suspected PPD than those who lived with their parents or parents-in-law as an extended family. Insufficient support by partners, parents, and parents-in-law was also associated with suspected PPD in this study. These findings were similar to those of previous studies conducted in Myanmar and other countries [7,11,31]. Support from partners and family members are important for mothers during the postpartum period due to their close proximity. These results suggest that healthcare providers should provide education about PPD to partners and family members while taking familial settings into account.

In this study, women who had depressive symptoms during the last pregnancy were significantly more likely to have suspected PPD than those who did not. This result is in line with the results of previous studies that found PPD was considerably more prevalent among women who experienced antenatal depression [2,9,34,39]. If depression is not detected or treated in the first or second trimester, and may persist until the third trimester, depression in the third trimester is strongly associated with PPD [4]. Pre-delivery factors on pain other than psychological statuses also may contribute to developing PPD, such as labor pain experience, pain catastrophizing, and anxiety. PPD is not commonly recognized in Myanmar; therefore, many women do not visit health facilities for treatment [17]. Women must be aware of the symptoms of depression that affect them, their children, and their families. Healthcare providers should provide information about PPD to pregnant and postpartum women to help them to understand about PPD. Healthcare providers should also identify pregnant and postpartum women at high risk of PPD and refer them to psychiatric specialists.

This study has some limitations. First, the study was conducted in a tertiary hospital in Myanmar, which has a higher prevalence of skilled birth attendance and hospital-based delivery than that in other urban areas. As a consequence, the results of this study may not represent the data on women in urban areas nationwide. Second, this study is a cross-sectional study; therefore, a cause-and-effect relationship between PPD and variables cannot be examined. Third, suspected PPD defined by the EPDS was used in this study, but not depression diagnosed by psychiatric specialists based on the Diagnostics and Statistical Manual of Mental Disorders (DSM-5) [25]. However, the Myanmar version of EPDS was validated using a clinical diagnosis of depression [20].

## 5 Conclusion

The prevalence of suspected PPD was 31.9 % among 6–8 weeks postpartum women in a tertiary hospital in Yangon, Myanmar. Factors associated with suspected PPD were having a nuclear family, a feeling of insufficient income, having an unplanned pregnancy, less than eight ANC visits, LBW babies, no support by partners, parents, and parents-in-law, and depressive symptoms during the last pregnancy. Strengthening maternal health programs, particularly family planning, antenatal care, and postpartum care, is crucial. It is essential to provide education about postpartum psychiatric problems not only to pregnant women but also to their families and health professionals to identify women who need psychosocial support. In order to improve maternal psychological well-being and mental health, the development and implementation of a comprehensive strategy that involves PPD screening and treatment in postpartum care is needed.

## Funding

This research received no external funding.

## Ethics approval

This study was approved by the Ethics Committee of the Myanmar Ministry of Health (approval number: NOGTH/2/2022). Written informed consent was obtained from each participant.

## Data availability statement

Data will be made available on request.

## CRediT authorship contribution statement

**Kaung Myat Wyunn:** Writing – review & editing, Writing – original draft, Visualization, Software, Project administration, Methodology, Formal analysis, Data curation, Conceptualization. **Zin Zin Than Wai:** Writing – review & editing, Resources, Methodology, Formal analysis, Data curation. **Khin Ei Ei Swe:** Writing – review & editing, Resources, Methodology, Formal analysis, Data curation. **Souphalak Inthaphatha:** Writing – review & editing, Formal analysis. **Kimihiro Nishino:** Writing – review & editing, Formal analysis. **Eiko Yamamoto:** Writing – review & editing, Supervision, Methodology, Formal analysis, Conceptualization.

## Declaration of competing interest

The authors declare that they have no known competing financial interests or personal relationships that could have appeared to influence the work reported in this paper.
