# Supplementary material for: Factors associated with postpartum depression among Myanmar women in Yangon: A cross-sectional study
Source: Heliyon. 2024 Jun 25;10(13):e33425. doi: 10.1016/j.heliyon.2024.e33425 (PMC11261082; doi:10.1016/j.heliyon.2024.e33425)
Supplement: Multimedia component 1 [file mmc1.docx]

Supplementary Table 1 Questionnaire to postpartum women

| No | Questions | Answer |
| --- | --- | --- |
| 1 | Age | ------------years old |
| 2 | Residence | Township -----------------------  State/region ----------------------- |
| 3 | Occupation | 1. Housewife 2. Student 3. Manual labor 4. Company staff 5. Government staff 6. Own business 7. Other --------------------- |
| 4 | Ethnic group |  |
| 5 | Religion | 1. Buddhist 2. Christian 3. Islam 4. Hindu 5. Other --------------------- |
| 6 | Marital status | 1. Single 2. Married 3. Divorced/separated 4. Widowed 5. Other --------------------- |
| 7 | Educational level (completed school) | 1. illiterate 2. Only read and write 3. Primary school 4. Middle school 5. High school 6. University 7. Graduate and above |
| 8 | Type of family | 1. Nuclear family 2. Extended family (living with parents or parents-in-law) |
| 9 | Household income per month (kyats) | 1. < 100,000 2. 100,000 - 300,000 3. 300,001 - 500,000 4. 500,001 - 700,000 5. 700,001 - 900,000 6. > 900,000 |
| 10 | Is the household income enough for your family’s living? | 1. Not at all 2. Yes |
| 11 | Partner’s/husband’s occupation | 1. Student 2. Manual labor 3. Company staff 4. Government staff 5. Own business 6. Other --------------------- |
| 12 | Partner’s/husband’s educational level  (completed school) | 1. Illiterate 2. Only read and write 3. Primary school 4. Middle school 5. High school 6. University 7. Graduate and above |
| 13 | Current relationship with your partner/husband | 1. Very good and happy 2. Good 3. Neutral 4. Bad, quarreling is often 5. Very bad, I want to get divorced. |
| 14 | Does your partner/husband have a history of medical diseases including depression? | 1. No, he doesn’t. 2. Yes, he does. 3. I don’t know. |
| 15 | Number of pregnancies |  |
| 16 | Number of deliveries |  |
| 17 | Do you have a history of miscarriages? If yes, how many times? | 1. No 2. Yes, I had ------------- times |
| 18 | How old is the last child? | ------ months old |
| 19 | Sex of the last child | 1. Male 2. Female |
| 20 | Was the last pregnancy unplanned or planned? | 1. Unplanned 2. Planned |
| 21 | Did you have antenatal care visits during the last pregnancy? If yes, how many times? | 1. No 2. Yes, I had ------------- times |
| 22 | Desired sex of the baby | 1. Desired 2. Undesired 3. I don’t mind. |
| 23 | Date of delivery of the last child | -----/-----/----- (DD/MM/YY) |
| 24 | Mode of delivery of the last child | 1. Spontaneous vaginal delivery 2. Assisted vaginal delivery   (instrumental delivery)   1. Emergency cesarean section 2. Elective cesarean section |
| 25 | Place of delivery of the last child | 1. Home 2. Private hospital/clinic 3. Primary health center 4. Public hospital 5. Other -------------------- |
| 26 | Who assisted when you delivered the last baby? | 1. Doctor 2. Nurse/midwife 3. Traditional birth attendant 4. Other ------------------------- |
| 27 | Did you have any illness during the last pregnancy? | 1. No 2. Yes --------------------- |
| 28 | Did your baby have any complications or problems after birth or stay at a hospital? | 1. No 2. Yes --------------------- |
| 29 | Gestational age of the last baby | ------------- weeks |
| 30 | Birth weight of the last baby | ------------- g |
| 31 | Is the born infant alive until now? | 1. No 2. Yes |
| 32 | Breastfeeding status | 1. Only breastmilk 2. Mixed (breastmilk + baby formula) 3. Baby formula |
| 33 | How was support from your husband/partner during your pregnancy and delivery? | 1. Very good 2. Good 3. Nothing good, nothing bad 4. Not good 5. Very bad 6. Other --------------------------- |
| 34 | How was support from your parents, especially your mother, during your pregnancy and delivery? | 1. Very good 2. Good 3. Nothing good, nothing bad 4. Not good 5. Very bad 6. Other -------------------------- |
| 35 | How was support from your parents-in-law, especially your mother-in-law, during your pregnancy and delivery? | 1. Very good 2. Good 3. Nothing good, nothing bad 4. Not good 5. Very bad 6. Other -------------------------- |
| 36 | How was support from your friends during your pregnancy and delivery? | 1. Very good 2. Good 3. Nothing good, nothing bad 4. Not good 5. Very bad 6. Other -------------------------- |
| 38 | Did you have any disease or receive treatment before the last pregnancy? | 1. No 2. Yes -------------------------- |
| 39 | Have you experienced the following symptoms (A-D) for 2 weeks or more before the last pregnancy?  (A) Depressed mode (feel sad, hopeless, tearful) nearly every day  (B) Markedly loss of interest (or pleasure) nearly every day  (C) Appetite problem (eat too much or eat too little), sleeping problem (insomnia or hypersomnia), loss of energy, or feelings of worthlessness nearly every day  (D) Recurrent thoughts of death | 1. No 2. Yes |
| 40 | Have you experienced the above symptoms (A-D) for 2 weeks or more during the last pregnancy? | 1. No 2. Yes |
| 41 | Have you taken any antidepressant or psychotropic drugs? | 1. No 2. Yes |
| 42 | Are you taking any antidepressant or psychotropic drugs now? | 1. No 2. Yes |
